# New strategy to control cell migration and metastasis regulated by CCN2/CTGF

**DOI:** 10.1186/1475-2867-14-61

**Published:** 2014-07-12

**Authors:** Diego Pinheiro Aguiar, Gabriel Correa de Farias, Eduardo Branco de Sousa, Juliana de Mattos Coelho-Aguiar, Julie Calixto Lobo, Priscila Ladeira Casado, Maria Eugênia Leite Duarte, José Garcia Ribeiro Abreu

**Affiliations:** 1 Research Division, National Institute of Traumatology and Orthopedics, Rio de Janeiro, RJ, Brazil; 2 Program of Cell and Developmental Biology, Institute of Biomedical Sciences, Federal University of Rio de Janeiro, Rio de Janeiro, RJ, Brazil

**Keywords:** CCN2, CTGF, Adhesion, Migration and Metastasis

## Abstract

Connective tissue growth factor (CTGF)/CCN family member 2 (CCN2) is a CCN family member of matricellular signaling modulators. It has been shown that CCN2/CTGF mediates cell adhesion, aggregation and migration in a large variety of cell types, including vascular endothelial cells, fibroblasts, epithelial cells, aortic smooth muscle and also pluripotent stem cells. Others matricellular proteins are capable of interacting with CCN2/CTGF to mediate its function. Cell migration is a key feature for tumor cell invasion and metastasis. CCN2/CTGF seems to be a prognostic marker for cancer. In addition, here we intend to discuss recent discoveries and a new strategy to develop therapies against CCN2/CTGF, in order to treat cancer metastasis.

## CCN2/CTGF historical profile

**Figure 1:**
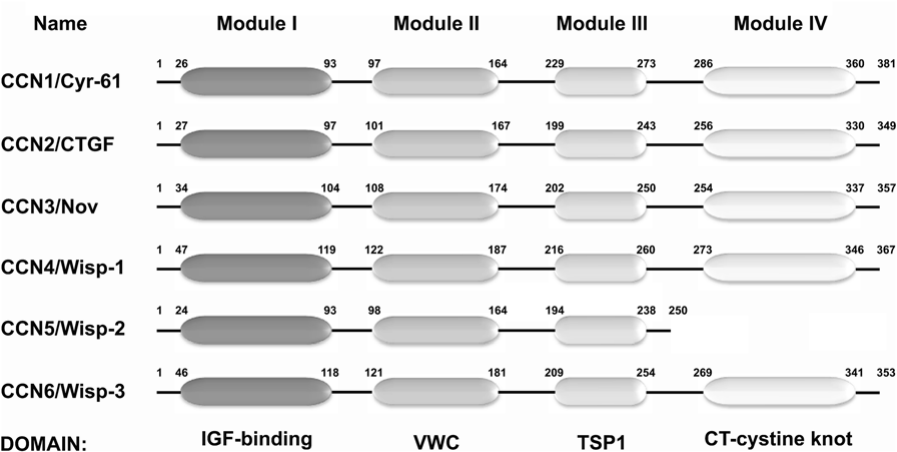
**Schematic representation of the CCN family structure.** The figure shows the current nomenclature (CCN family) and the most used name along the last years. The CCN family domains are arranged into modules as: IGF-binding/module I, VWC/module II, TSP1/module II and CT-cystine knot/module IV. The size in each domain of each CCN family member related to their biological functions [11-14].

Connective tissue growth factor (CTGF)/CCN2 family member 2 (CCN2) was isolated from human endothelial cells and outlines a secreted growth factor involved in cell proliferation and chemotaxis as former described in 1991, by Dr Bradham et al. [1]. CCN2/CTGF has 349 amino acids and depending on post-translational modifications, CCN2/CTGF can be glycosylated, causing either 36 or 38 kDa, which appears as a double band on Western immunoblotting [2]. In the same year, CCN2/CTGF orthologous was described in mice and named Fisp-12 [3] or even other synonyms as βIG-M2, IGF-BP8, IGFBP-rP2, HBGF-0.8, Hcs24 or ecogenin [4]. Since its identification, CCN2/CTGF’s role has been studied, in a large variety of biological phenomena, especially in cell chemotaxis, migration, adhesion and cancer. Still in the nineties, other proteins with the same domains were identified, such as cysteine-rich 61 (Cyr61), nephroblastoma overexpressed (Nov), expressed in low-metastatic protein (Elm1/Wisp-1), WNT1-inducible-signaling pathway protein-2 (Wisp-2) and WNT1-inducible-signaling pathway protein-3 (Wisp-3) [5-9]. In the following decade, researches who have had contributed to the understanding of these proteins, proposed the unification name of these secreted factors in the CCN family (**C**YR61/**C**TGF/**N**OV), then called CCN1 (Cyr61), CCN2 (CTGF), CCN3 (Nov), CCN4 (Wisp-1), CCN5 (Wisp-2), and CCN6 (Wisp-3) [10]. These proteins share a multimodular structure, with a N-terminal secretory signal followed by four conserved domains with homologies to insulin-like growth factor binding proteins (Module I IGFBP), von willebrand factor type C repeat (module II VWC), thrombospondin type I repeat (Module III TSP) and a carboxy-terminal domain (Module IV - CT) containing a cystine knot [absent in CCN5] [10] (Figure 1).

Studies about the CCN family have been intensified in the last years mainly to understand CCN2/CTGF function. Several studies had also shown the relevance of each catalytic domain on proliferation and chemotaxis [1], cell fate [15], adhesion [16], and migration [17] in different cell types and tissues. Several publications had also described interactions of the CCN2/CTGF with extracellular matrix proteins [18]. Ivkovic et al. in 2003 developed a knockout mouse for CCN2/CTGF showing its role in extracellular matrix remodeling and angiogenesis during chondrogenesis [19]. In addition, CCN2/CTGF knockout mice also shows lower expression of metalloproteinase 9 and vascular endothelial growth factor (VEGF) in the growth plate. These changes lead to skeletal dimorphism, with expanded hypertrophic zones of *CTGF* mutant growth plates and a defective replacement of cartilage by bone during endochondral ossification. This study was the first to demonstrate the role of CCN2/CTGF during development.

### CCN2/CTGF in cell adhesion and migration

The synthesis of CCN2/CTGF is highly inducible by serum growth factors [20], cytokines [21], and environmental stresses such as hypoxia [22] and molecular stretch [23]. Well known inducers of CCN2/CTGF are transforming growth factor beta (TGF-beta) and VEGF [24,25].

**Figure 2:**
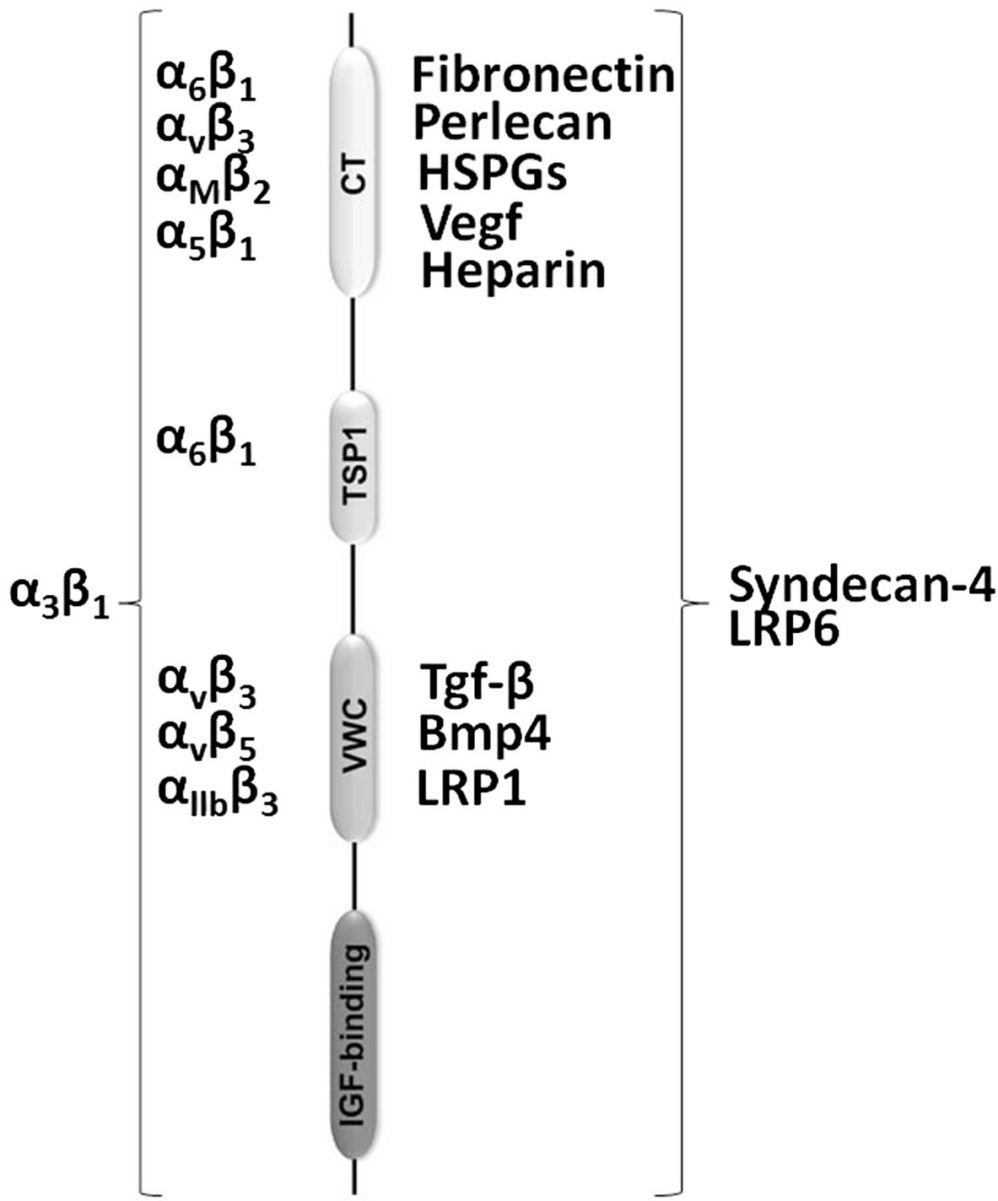
**Interaction of CCN2/CTGF domains with other molecules.** The right column shows the interaction between CCN2/CTGF with cell surface receptors, extracellular matrix and growth factors, as Fibronectin, Perlecan, HSPGs, VEGF, TGF-β, BMP4, LRP1, Heparin, Syndecan-4 [28,29,31-35]. The left column shows the interaction between CCN2/CTGF with integrins [30,36-41].

**Figure 3:**
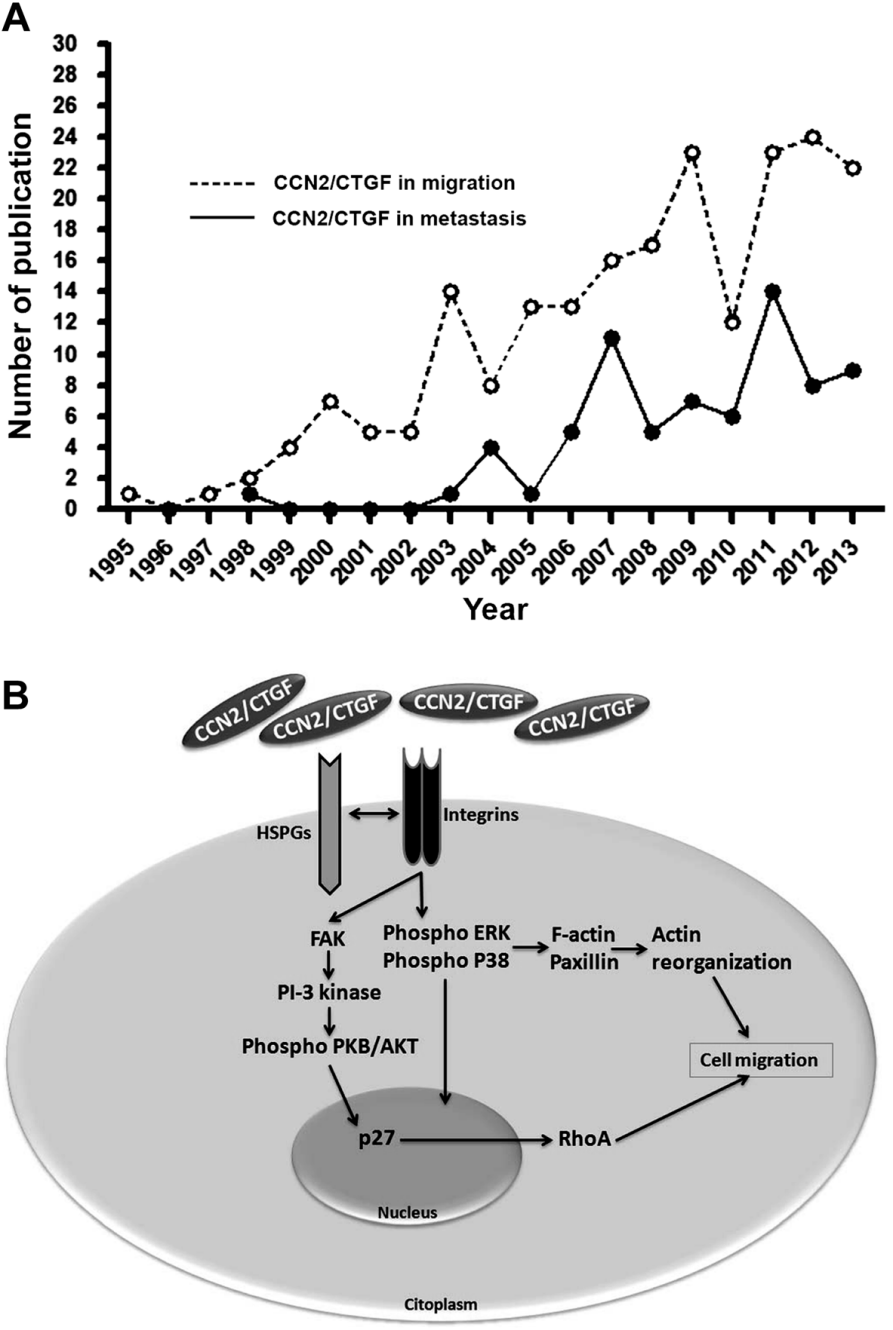
**Impact of CCN2/CTGF research in cancer metastasis. (A)** Evaluation number of articles published per year that studied the role of CCN2/CTGF on cell migration and metastasis **(B)** Cell migration and adhesion modulated by CCN2/CTGF. CCN2/CTGF promotes cells migration and adhesion by binding to HSPGs and Integrins in cell surface. Integrins activation potentiates the phosphorylation of the ERK and P38, leading to activation of F-actin and Paxillin resulting in actin reorganization [42]. The same pathway can stimulate FAK, PI-3 kinase ensuing phosphorylation of the PKB/AKT and translocations of P27 to the nucleus that promotes the transcriptional control of RhoA, and thus enhances focal adhesion formation and cellular migration [32].

Despite researches willingness, a specific receptor for CCN2/CTGF was never described. However, several possible receptors for this growth factor had been investigated in CCN2/CTGF-mediated adhesion, migration and chemotaxis, including integrins (α_6_β_1_,α_v_β_3,_α_M_β_2,_α_5_β_1,_α_6_β_1,_α_v_β_3,_α_v_β_5,_α_IIb_β_3_ and α_3_β_1_) [26,27], LRP1 and LRP6 [28,29], and HSPGs (co-receptor) [30] on cell membrane (Figure 2). There are currently 89 published papers relating CCN2/CTGF to migration and adhesion, however, no one has established a CCN2/CTGF receptor (Figure 3A).

The multiple functions of CCN2/CTGF may be explained in part by its interactions with other molecules in the extracellular domain. CCN2/CTGF binds to TGF-beta through the VWC domain and enhances its binding to TGF-beta receptor II, increasing its signaling [31,43]. A comparison of a pull of mRNA extracted from wild type mouse embryonic fibroblast (MEFs) to MEFs deleted for CCN2/CTGF(CTGF-/- MEF), both exposed to TGF-beta, showed downregulation of several molecules involved in matrix production and remodeling, cell adhesion and contraction, such as: Filamin-β, β-Catenin and matrix metalloproteinase-14. These results showed CCN2/CTGF as a cofactor required to active cell adhesion by TGF-beta [44]. Since CCN2/CTGF was described to interact with other extracellular matrix proteins [45], many efforts have been made to understand how can CCN2/CTGF acts in cell adhesion and migration.

An efficient way to show the role of CCN2/CTGF in cell adhesion was through the exposure of different cell types to a CCN2/CTGF-rich substrate. Thus, it was possible to show that cells exposed to CCN2/CTGF adhered to substrate faster than cells exposed only to free-CCN2/CTGF substrate [46,47]. In order to assay the role of CCN2/CTGF in cell adhesion, CCN2/CTGF coated plastic surface and anti-CCN2/CTGF antibody were used to measure cell adhesion of four different cells types: vascular endothelial cells [HUVECs and human microvascular endothelial cells (HMVECs)], fibroblasts (NIH 3T3 and AKR2B), mink lung epithelial (Mv1Lu) cells [26,45], and human platelets [48]. All results showed that the absence of CCN2/CTGF prevents cell adhesion. In addition, the CCN2/CTGF-mediated adhesion occurs through interaction with other molecules, as fibronectin and integrins, reviewed by Arnott et al in 2011 [48]. CCN2/CTGF CT-domain interacts with fibronectin and enhances cell adhesion of chondrocytes through integrin alpha5beta1 [49]. CCN2/CTGF promotes fibroblast adhesion by binding to fibronectin, cell surface proteoglycans, and integrins that potentiates the phosphorylation on focal adhesion Kinase (FAK) and ERK and so enhances focal adhesion formation and cell spreading by F-actin, Paxilin and RhoA activation [32] (Figure 3B). Although these data suggested that CCN2/CTGF is an adhesive molecule, this property was never directly tested. However, a recent work of our research group demonstrated that CCN2/CTGF is not an adhesive molecule itself.

For the first time optical tweezers technique was used to measure the adhesion strength of different molecules. Initially, It was observed that CCN2/CTGF induced spherical cell aggregates formation when added to MvLu1 and P19 cells [31,46,48]. A dynamic system to assay cell aggregation to CCN2/CTGF was developed by using anti-Flag conjugated agarose beads pre-incubated with recombinant CCN2/CTGF enriched medium and added to sub-confluent P19 cells cultured for 24 h. This experiment aimed to check the local action of CCN2/CTGF on P19 cells aggregation. It was observed none or few P19 cells interacting with untreated beads. In contrast, many P19 cells were detected surrounding and adhering to CCN2/CTGF-treated beads. The attached cells displayed morphological changes varying from a flat to an elongated shape reminiscent from the bead shape [46,48]. In this study, Boyden chamber assay indicated CCN2/CTGF as a chemoattractive molecule increasing the migration of P19 cells up to three folds [48]. In the same study, a new approach was used to measure the adhesive potential of CCN2/CTGF and to compare it with well known adhesive molecules. Using optical tweezers, we obtained quantitative parameters to evaluate molecular adhesiveness. Our data supported the CCN2/CTGF chemotactic property, although it is not an adhesive molecule for P19 cells.

### From innocent to guilty: CCN2/CTGF as a key molecule in metastasis

The link between CCN2/CTGF and cell adhesion is based on protein interactions in extracellular domain, stimulus in extracellular matrix production and upregulation of adhesion pathways, but not due an own adhesive property [48]. These adhesion pathways are triggered by Integrins or MAPK activation resulting in cell attachment and detachment [50]. Knockdown of CCN2/CTGF in TW2.6 cells was shown to reduce tumor formation and decrease E-cadherin expression in xenotransplanted tumors [51]. Although there has been a huge variety of research conducted on the harmful effects of increasing levels of CCN2/CTGF, the understanding of its pathophysiology and possible modulators remain unclear. Deendooven et al. detected CCN2/CTGF levels in plasma, serum or urine in patients with fibrotic complications in consequence of hepatitis, diabetes and renal transplantation [52]. The high levels of CCN2/CTGF in these secretions suggested that it is a relevant biomarker of disorders. Furthermore, there are no efficient treatments for most disorders where the upregulation of CCN2/CTGF is involved.

Taking into account the role of CCN2/CTGF on cell migration process, the expression of CCN2/CTGF has been correlated to metastasis progression. Overexpression of CCN2/CTGF was associated with invasiveness potential of the lung adenocarcinoma cells [53], and osteolytic metastasis of breast cancer [54]. Interestingly, CCN2/CTGF enhanced the motility of breast cancer cells by an integrin-α5β3-ERK1/2-phosphorylation [42]. In chondrosarcoma cells, CCN2/CTGF enhances cell migration by matrix metalloproteinase-13 upregulation through integrin α5β3 [55] and in gastric cancer through downregulation of E-cadherin by NF-κB pathway [56]. Curiously, neutralizing anti-CCN2/CTGF antibody attenuated metastasis pancreatic cancer [57]. In cancer and in metastasis, the role of CCN2/CTGF seems to be the same. In the last years, the number of studies showing CCN2/CTGF involved in migration and metastasis has increased. The treatment of patients with metastasis is not efficient. For this reason, the occurrence of metastasis in cancer is an important issue nowadays, and CCN2/CTGF could be an important target for its attenuation or prevention.

### CCN2/CTGF a challenge for the future: Where will we go?

**Figure 4:**
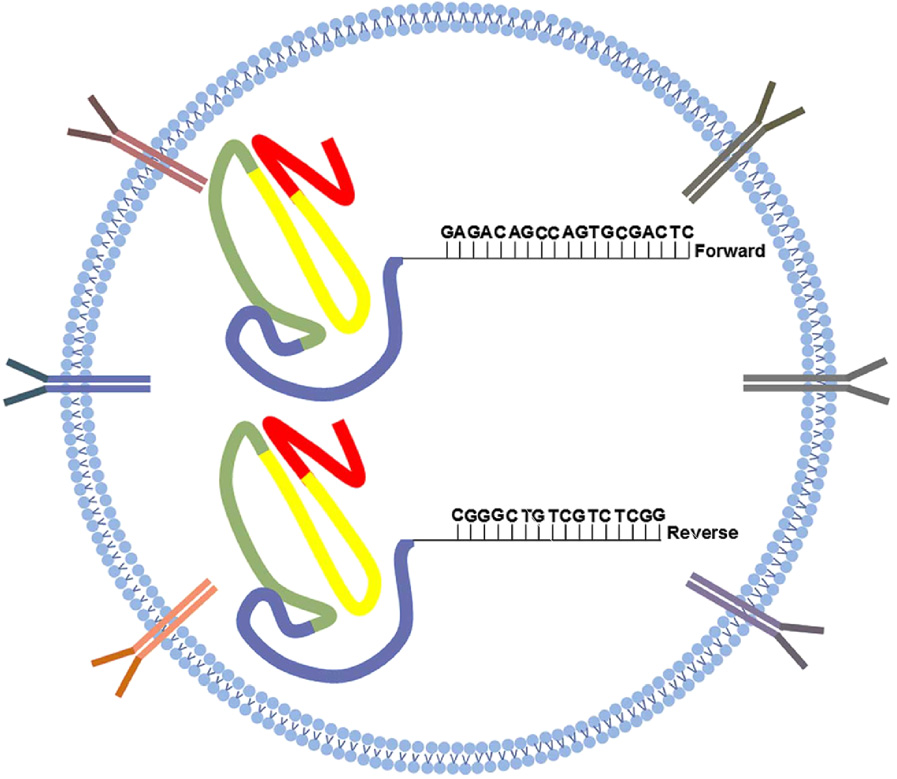
**Repression of CCN2/CTGF transcription by methylation.** Antibodies distributed over the membrane of the liposome vesicle, could be used to recognize target cells as: Ovarian tumor cells, breast tumor cells or hiperproliferative fibroblast during wound healing. It might allow more specificity in the blockage. The DNA methyl-transferase enzyme linked to specific primer for CCN2/CTGF promoter region is located inside the lipofection body. The rational allow us to imagine that CCN2/CTGF gene hipermethylation will promote its downexpression. CCN2/CTGF Primers forward 5′ GAGACAGCCAGTGCGACTC 3′ Reverse 3′ CGGGCTGTCGTCTCGG 5′.

In order to develop specific treatments for disorders where CCN2/CTGF are upregulated, as metastasis, CCN2/CTGF shutdown by epigenetic modulation may be an effective strategy. In general, DNA methylation patterns are established and modified in response to environmental factors by DNA methyl-transferases (DNMTs) which inhibits gene transcription and regulates gene expression [58]. Therefore, inhibition of CCN2/CTGF expression in specific diseases could be achieved by increasing DNA methylation of the CCN2/CTGF gene on promoter region. DNA methylation of the CCN2/CTGF genomic sequence has already been reported in several cancers [59,60], but a detailed target sequence for DNA methylation contributing for gene silencing had never been described. The mechanisms of CCN2/CTGF expression remain unknown. The delivery of DNA methyl-transferase enzyme linked to specific primer for CCN2/CTGF in promoter region directs to the target cell, through antibodies that recognize specifically target cell, could be explored as a way to silence CCN2/CTGF. This blockage-pack would have a DNMT linked to CCN2/CTGF primers (Forward and Reverse sense) designed to its promoter region. DNMT would hypermethylate the CCN2/CTGF promoter region and CpG islands will be increased leading to CCN2/CTGF donwregulation (Figure 4). A homozygous deletion of CCN2/CTGF at human chromosome 6q23.2 by hypermethylation could be used or breast cancer cells, for example. It would be useful to block its expression and then decrease cancer metastasis. Our proposed DNA methylation strategy on promoter region of CCN2/CTGF gene to control its expression would certainly control all chain reactions triggered by this growth factor. We hope that in the future, alike strategies that could be used to block target gene transcription and, consequently, inhibit its migration and adhesion by preventing several disorders as metastasis, fibrosis or tissue remodeling.

## Competing interests

The authors declare that they have no competing interests.

## Authors' contributions

DPA and JGRAJr conceived the idea. DPA and JMCA integrated different points of literatures and drafted the manuscript. GCF, EBS, JCL, PLC, and MELD did literature research on specific points, and got involved in discussion. All authors read and approved the final manuscript.
